# Increased Risk of Stillbirth among Women whose Partner Has Tuberculosis

**DOI:** 10.1155/2021/1837881

**Published:** 2021-09-14

**Authors:** Qi Sun, Hongguang Zhang, Ya Zhang, Zuoqi Peng, Jianbo Lu, Xu Ma

**Affiliations:** ^1^Human Genetics Resource Center, National Research Institute for Family Planning, Beijing 100081, China; ^2^Graduate School of Peking Union Medical College & Chinese Academy of Medical Sciences, Beijing 100730, China

## Abstract

**Background:**

The relationship between tuberculosis (TB) and adverse pregnancy outcomes remains unclear. The aim of our study was to investigate whether TB is a risk factor for adverse pregnancy outcomes including premature birth, low birth weight, and stillbirth.

**Method:**

We conducted a population-based retrospective cohort study in mainland China. A total of 3,668,004 Chinese women, along with their partners, were included in this study, within the National Free Pre-Pregnancy Checkups Project, during 2015–2018. Propensity score matching was used to balance the two groups (cases: women or partners with TB; controls: women and partners without TB). Multivariable logistic regression was used to estimate odds ratios (ORs) and 95% confidence intervals (CIs).

**Results:**

Multivariate logistic regression showed that the OR of stillbirth for cases was 1.89 (95% CI: 1.09–3.16), in comparison with the control group. In the subgroup analysis, women whose partner had TB had a higher risk of stillbirth (OR: 2.13, 95% CI: 1.10–3.86) than women whose partner did not have TB. There was no significant difference in adverse pregnancy outcomes, including preterm birth, low birth weight, and stillbirth, between women with and without TB.

**Conclusions:**

Women whose partner had TB were more likely to have stillbirth than women whose partners did not have TB.

## 1. Background

Tuberculosis (TB) continues to be a major public health problem, causing 1.5 million deaths in 2018, and it is the one of the 10 leading causes of death [1]. In 2018, the World Health Organization (WHO) reported that 44% of TB cases occurred in regions of Southeast Asia, with 9% of cases being diagnosed in China [2]. TB not only has high mortality, but it also leads to considerable short- and long-term health consequences [3]. Although new drugs and vaccines have been developed to reduce the burden of TB, this disease remains a serious global health concern.

TB mainly affects the lung tissue of patients, but it can also spread to other organs of the body [4]. The third most common form of extrapulmonary TB is female genital TB, which can spread from the lungs through the blood [5]. *Mycobacterium tuberculosis* affects the endometrium, fallopian tubes, ovaries, and so on, leading to infertility [6]. Studies have found that TB can lead to adverse pregnancy outcomes, including premature birth, low birth weight (LBW), stillbirth, and cesarean delivery [6]. However, there are some inconsistencies among the relevant study findings. Recent evidence from the United States suggests that women with TB are more likely to experience postpartum anemia, preterm birth (PTB), and pneumonia than women without TB; in that study, the incidence of maternal respiratory complications were also higher in the TB group than in the control group [7]. A study conducted in Taiwan also showed that women with TB were at increased risk for adverse pregnancy outcomes compared with controls [8]. Another study found that the percentages of LBW and having a short gestational age were higher among women diagnosed with TB than in women without TB, but there was no significant difference in PTB between the two groups [8]. However, a prospective study in India suggested that there were no statistically significant differences in pregnancy outcomes between women with TB and controls [9]. These inconsistent results might be owing to the limited sample sizes, insufficient control of confounding factors, and ethnicity in these studies.

Generally, studies have examined the influence of female factors on adverse pregnancy outcomes; however, little research has been focused on the role of partners in adverse pregnancy outcomes. In our previous study, we found associations of smoking among partners with the prevalence of hypertension among women [10]. Moreover, a case study reported that a healthy asymptomatic woman had been infected with genital TB via sexual intercourse with her partner [11]. Therefore, it is necessary to assess the TB status of partners when investigating the relationship between TB and adverse pregnancy outcomes in women.

The main purpose of this study was to investigate the relationship between TB in women and adverse pregnancy outcomes in mainland China. A second study aim was to investigate the influence of TB in partners on women's adverse pregnancy outcomes.

## 2. Methods

### 2.1. Study Population

**Figure 1:**
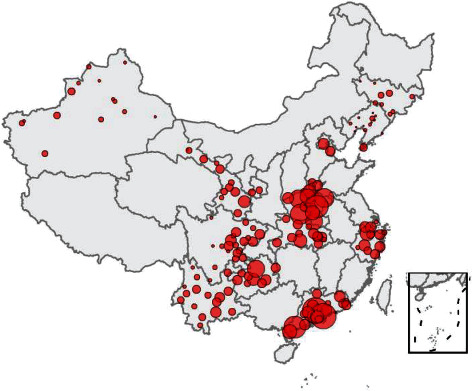
The spatial distribution of cities was included in the study (this statistical map was drawn by ourselves using R software 4.0.0).

All data included in this study were obtained from the National Free Pre-Pregnancy Checkups Project (NFPCP). The NFPCP was supported by the National Health and Family Planning Commission and the Ministry of Finance of the People's Republic of China. Free prepregnancy medical examinations and counseling services are provided by the NFPCP to reproductive couples, and most Chinese cities are included in this project. In our study, we included 164 cities in 14 provinces of China from 2015 to 2018 (Figure 1).

All participants included in the NFPCP were asked to complete a structured questionnaire that included information on maternal characteristics, education, alcohol consumption, and smoking. Physical examinations and measurements including weight, height, and blood pressure were conducted by a local doctor. Pregnancy outcomes were obtained during follow-up. TB status was reported by participants in face-to-face interviews.

### 2.2. Pregnancy Outcomes

Adverse pregnancy outcomes in our study included preterm birth (PTB), low birth weight (LBW), and stillbirth. PTB is defined as an infant born at less than 37 weeks' gestation [12]. LBW is defined as an infant with birth weight less than 2500 g, according to the WHO [13]. Stillbirth is defined as fetal death at or after 20 or 28 weeks of pregnancy [14].

### 2.3. Covariates

We obtained information on disease history, eating habits, lifestyle, and results of physical and laboratory examinations for pregnant women and their partners. Education was divided into three levels: junior high school and below, high school and junior college, and university and above. Intensity of work was divided into three levels including light, moderate, and heavy, according to participants' occupation. Smoking was defined as smoking two cigarettes a day.

### 2.4. Statistical Analysis

The Kolmogorov–Smirnov test was used to assess the normality of variables. For continuous variables, *t*-tests or Mann–Whitney *U* tests were performed, depending on their distribution. The chi-square test was used to compare the differences in categorical variables. Propensity score matching (PSM) using body mass index and age was used to balance the two groups. Multiple imputations were applied to impute variables with missing values, with less than 10% of variables missing. To study the association between TB exposure and adverse pregnancy outcomes, we used a logistic regression model. If the *P* value of a variable in the univariate analysis was less than 0.05, the variable was included in the multivariate analysis. All analyses were performed using R software version 4.0.0 (The R Project for Statistical Computing). A two-sided *P* value < 0.05 was considered statistically significant for all statistical tests in our study.

## 3. Results

**Figure 2:**
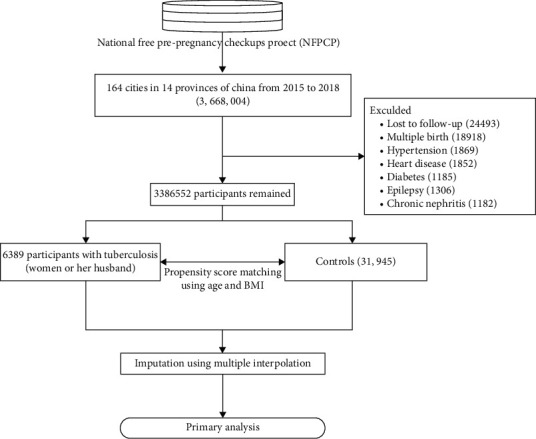
Flowchart of our study.

A total of 3,668,004 women and their partners were included in this study, after excluding individuals with multiple pregnancies, hypertension, heart diseases, diabetes, epilepsy, and chronic nephritis. PSM at a ratio 1 : 5 was used to minimize potential bias owing to unequal distribution between the two groups (cases: women or partners with TB; controls: couples without TB). Details of the study flow are given in Figure 2.

There were significant differences between the case group and the control group with respect to the characteristics of women and partners, including ethnicity, education level, work intensity, residence, and regularity of menstrual cycles (all *P* < 0.05). There were no significant differences between cases and controls in terms of age at menarche, eating habits, and blood pressure, among others (Table 1). We performed two subgroup analyses (subgroup 1: women with and without TB; subgroup 2: partners with and without TB). The results of the subgroup analyses are shown in Supplementary Table S1.

The outcomes of pregnancy between the case and control groups are shown in Table 2. Higher percentages of PTB (2.4% vs. 1.9%, *P* = 0.01), LBW (2.0% vs. 1.5%, *P* < 0.01), and stillbirth (0.3% vs. 0.1%, *P* < 0.01) were observed among cases in comparison with controls (Table 2). Moreover, we identified a significant difference in LBW between women with and without TB and in PTB and stillbirth between partners with and without TB (Supplementary Table S1).

Multivariate logistic regression showed that the odds ratio (OR) of stillbirth for cases was 1.89 (95% confidence interval (CI): 1.09–3.16), compared with the control group. However, there were no significant differences in PTB and LBW between cases and controls (Table 3). In the subgroup analysis, we found no significant differences in PTB (OR: 0.87, 95% CI: 0.66–1.12), stillbirth (OR: 1.48, 95% CI: 0.61–3.09), and LBW (OR: 1.04, 95% CI: 0.79–1.35) between women with and without TB (Table 3). Women whose partner had TB had a higher risk of stillbirth (OR: 2.13, 95% CI: 1.10–3.86) than women whose partner did not have TB (Table 3). Regarding the treatment of tuberculosis, 39 pregnant women self-reported that they were taking one of the antituberculosis drugs including isoniazid, rifampicin, ethambutol, and pyrazinamide. In order to avoid the influence of drug treatment on pregnancy outcome, we excluded pregnant women undergoing antituberculosis treatment, and the results are consistent with the main analysis (Supplementary Table S2).

## 4. Discussion

This nationwide study included 14 provinces and 164 cities of China, and the sampling sites were located throughout most parts of China. We demonstrated that if a woman or her partner had TB, the woman had a greater risk of stillbirth. Moreover, in subgroup analysis, we found that women whose partner had TB were 2.13 times more likely to have stillbirth than those without TB. However, no effects on PTB, stillbirth, and LBW were found for women with TB.

A cross-sectional retrospective study conducted in Taiwan found no significant differences in PTB between women with and without TB [8]. Tripathy and Tripathy did not observe any adverse pregnancy outcomes in women with TB [9]. Our findings were consistent with those studies in that we found no differences between women with and without TB with respect to adverse pregnancy outcomes, including PTB, LBW, and stillbirth. Our study was based on national data covering the vast majority of the population in China, which makes our research findings convincing.

However, some results in our study were inconsistent with those of previous studies. Some research findings have shown that women with TB have a higher risk of LBW [8], PTB, and perinatal death [15, 16]. A study conducted in India found that pregnant women with TB had a five times higher risk of PTB than healthy women [17]. There are several possible reasons for the inconsistencies between our research and previous studies including small sample size, few covariates, and different ethnic groups. In this study, our model incorporated lifestyle and dietary habits, disease history, and laboratory test results of pregnant women. Another important reason for the inconsistent results is that previous studies have not taken into account the effect of a woman's partner on pregnancy outcomes. Our previous study showed that this is important as we found an effect of the partner's smoking on spontaneous abortion [18].

The other main finding of the present study is that having a partner with TB was found to be a risk factor for stillbirth in pregnant women, which has not been reported previously. One case report demonstrated that TB can be transmitted from partner to his wife via semen [19]. Another study showed that female genital TB could influence endometrial metabolism [20]. These metabolic changes might be associated with stillbirth in pregnant women. Animal experiments have demonstrated that TB affects the histophysiology of the male reproductive system [21]. Another explanation for our result is that TB affects the quality of sperm, which could lead to stillbirth.

Previous studies have shown that TB treatment can reduce the risk of TB to pregnant women and fetuses [22]. Further studies found that TB treatment in the first trimester can reduce the risk of preterm birth, low birth weight, and fetal death [23]. In our study, antituberculosis drug treatment did not affect the pregnancy outcome of pregnant women. The possible reason for this result is that too few pregnant women in the cohort self-reported taking antituberculosis drugs. A possible reason for the low number of people taking antituberculosis drugs in our study was that all TB patients are assessed for suitability for pregnancy before conception. If a woman was taking antituberculosis drugs, her doctor may recommend delaying pregnancy until antituberculosis treatment was completed.

There were several limitations in this study that should be noted. First, in our study, TB status was self-reported, which made it impossible to confirm whether the participant's TB was active or not. Second, owing to self-reporting, some patients with TB might have concealed their medical history, which may lead to selection bias. Third, in the discussion, we hypothesized that the partner could transmit TB via semen, but we did not collect the quality of the partner's sperm, clinical condition of the partner at conception, and how long the couples have been in contact, which limited the reliability of our conjecture. Fourth, anti-TB treatment has an impact on pregnancy outcomes, but too few patients in our study were taking anti-TB drugs, which may have affected the results.

## 5. Conclusion

Among the pregnant women in our study, having TB was not a risk factor for LBW, PTB, and stillbirth. Women whose partners had TB were more likely to have stillbirth than women whose partners did not have TB.

## Figures and Tables

**Table 1:** Characteristics of the study population between cases and controls.

Variables		Control (31945)	Case (6389)	*P*
*Female's characteristics*				
Age (year)		28.00 [25.00, 31.00]	28.00 [25.00, 31.00]	0.937
Ethnicity	Han Chinese	29398 (92.0)	5752 (90.0)	<0.001
	Others	2547 (8.0)	637 (10.0)	
Education level	Junior high school and below	15471 (48.4)	2064 (32.3)	<0.001
	High school and junior college	14926 (46.7)	3711 (58.1)	
	Bachelor and above	1548 (4.8)	614 (9.6)	
Intensity of work	Light	6515 (20.4)	2180 (34.1)	<0.001
	Moderate	4492 (14.1)	1401 (21.9)	
	Heavy	20938 (65.5)	2808 (44.0)	
Residence	Country	26348 (82.5)	4339 (67.9)	<0.001
	City	5597 (17.5)	2050 (32.1)	
Menarche age		14.00 [13.00, 14.00]	13.00 [13.00, 14.00]	0.54
Has regular menstrual cycle	No	1729 (5.4)	655 (10.3)	<0.001
	Yes	30216 (94.6)	5734 (89.7)	
Menstrual blood volume	Much	819 (2.6)	209 (3.3)	<0.001
	Median	29815 (93.3)	5537 (86.7)	
	Few	1311 (4.1)	643 (10.1)	
Dysmenorrhea	No	22068 (69.1)	3528 (55.2)	<0.001
	Yes	9877 (30.9)	2861 (44.8)	
Gravidity	0	14970 (46.9)	2723 (42.6)	<0.001
	1	11946 (37.4)	2007 (31.4)	
	≥2	5029 (15.7)	1659 (26.0)	
Parity	0	16916 (53.0)	3787 (59.3)	<0.001
	≥1	15029 (47.0)	2602 (40.7)	
Eat meat and eggs regularly	No	276 (0.9)	62 (1.0)	0.449
	Yes	31669 (99.1)	6327 (99.0)	
Anorexia vegetables	No	31759 (99.4)	6349 (99.4)	0.743
	Yes	186 (0.6)	40 (0.6)	
Eat raw meat regularly	No	31806 (99.6)	6330 (99.1)	<0.001
	Yes	139 (0.4)	59 (0.9)	
Smoking	No	31854 (99.7)	6335 (99.2)	<0.001
	Yes	91 (0.3)	54 (0.8)	
Passive smoking	No	27747 (86.9)	4596 (71.9)	<0.001
	Yes	4198 (13.1)	1793 (28.1)	
Drinking	No	30697 (96.1)	5772 (90.3)	<0.001
	Yes	1248 (3.9)	617 (9.7)	
Pressure of life and work	Never	24511 (76.7)	3827 (59.9)	<0.001
	Mild	3994 (12.5)	922 (14.4)	
	Moderate	3166 (9.9)	1430 (22.4)	
	Severe	274 (0.9)	210 (3.3)	
BMI (kg/m^2^)		20.57 [19.04, 22.67]	20.56 [19.03, 22.67]	0.77
SBP (mmHg)		110.00 [100.00, 115.00]	110.00 [100.00, 116.00]	0.511
DBP (mmHg)		70.00 [66.00, 75.00]	70.00 [65.00, 76.00]	0.441
Blood group	O	10736 (33.6)	2370 (37.1)	<0.001
	A	9279 (29.0)	1807 (28.3)	
	B	9122 (28.6)	1738 (27.2)	
	AB	2808 (8.8)	474 (7.4)	
Rh blood group	Po	31843 (99.7)	6373 (99.7)	0.433
	Ne	102 (0.3)	16 (0.3)	
FBG (mmol/L)		4.78 [4.32, 5.20]	4.81 [4.40, 5.21]	<0.001
ALT (U/L)		16.00 [11.70, 22.90]	15.00 [11.00, 21.00]	<0.001
Creatinine (*μ*mol/L)		63.00 [53.00, 74.00]	59.00 [51.00, 70.00]	<0.001
TSH (mIU/L)		1.61 [1.07, 2.34]	1.78 [1.15, 2.65]	<0.001

*History of diseases in female*				
Anemia	No	31355 (98.2)	6133 (96.0)	<0.001
	Yes	590 (1.8)	256 (4.0)	
Thyroid disease	No	31739 (99.4)	6280 (98.3)	<0.001
	Yes	206 (0.6)	109 (1.7)	
History of premature birth	No	31867 (99.8)	6364 (99.6)	0.052
	Yes	78 (0.2)	25 (0.4)	
History of stillbirth	No	31693 (99.2)	6294 (98.5)	<0.001
	Yes	252 (0.8)	95 (1.5)	
History of natural abortion	No	30929 (96.8)	5956 (93.2)	<0.001
	Yes	1016 (3.2)	433 (6.8)	
History of artificial abortion	0	26664 (83.5)	4354 (68.1)	<0.001
	1	3605 (11.3)	1324 (20.7)	
	>1	1676 (5.2)	711 (11.1)	

*Partner's characteristics*				
Age (year)		29.00 [26.00, 33.00]	30.00 [27.00, 34.00]	<0.001
Ethnicity	Han	29537 (92.5)	5812 (91.0)	<0.001
	Other	2408 (7.5)	577 (9.0)	
Education level	Junior high school and below	15187 (47.5)	1992 (31.2)	<0.001
	High school and junior college	15185 (47.5)	3777 (59.1)	
	Bachelor and above	1573 (4.9)	620 (9.7)	
Intensity of work	Light	4974 (15.6)	1747 (27.3)	<0.001
	Moderate	5454 (17.1)	1651 (25.8)	
	Heavy	21517 (67.4)	2991 (46.8)	
Eat meat and eggs regularly	No	323 (1.0)	86 (1.3)	0.021
	Yes	31622 (99.0)	6303 (98.7)	
Anorexia vegetables	No	31765 (99.4)	6304 (98.7)	<0.001
	Yes	180 (0.6)	85 (1.3)	
Eat raw meat	No	31734 (99.3)	6299 (98.6)	<0.001
	Yes	211 (0.7)	90 (1.4)	
Smoking	No	22878 (71.6)	4182 (65.5)	<0.001
	Yes	9067 (28.4)	2207 (34.5)	
Passive smoking	No	23041 (72.1)	3487 (54.6)	<0.001
	Yes	8904 (27.9)	2902 (45.4)	
Drinking	No	22007 (68.9)	3266 (51.1)	<0.001
	Yes	9938 (31.1)	3123 (48.9)	
Pressure of life and work	Never	23371 (73.2)	3489 (54.6)	<0.001
	Mild	4723 (14.8)	960 (15.0)	
	Moderate	3370 (10.5)	1627 (25.5)	
	Severe	481 (1.5)	313 (4.9)	
BMI (kg/m^2^)		22.86 [21.00, 25.05]	22.49 [20.42, 24.77]	<0.001
SBP (mmHg)		118.00 [110.00, 121.00]	118.00 [110.00, 124.00]	<0.001
DBP (mmHg)		75.00 [70.00, 80.00]	75.00 [70.00, 80.00]	0.091
Blood group	O	10861 (34.0)	2335 (36.5)	<0.001
	A	9280 (29.0)	1870 (29.3)	
	B	9003 (28.2)	1705 (26.7)	
	AB	2801 (8.8)	479 (7.5)	
Rh blood group	Po	31839 (99.7)	6375 (99.8)	0.177
	Ne	106 (0.3)	14 (0.2)	
ALT (U/L)		25.00 [17.60, 36.00]	25.00 [17.00, 38.20]	0.114
Creatinine (*μ*mol/L)		81.00 [72.40, 91.00]	81.20 [72.50, 91.50]	0.484

The distribution of continuous variables is expressed as the median [lower quartile, upper quartile]. BMI: body mass index; SBP: systolic blood pressure; DBP: diastolic blood pressure; FBG: fasting blood glucose; ALT: alanine aminotransferase; TSH: thyroid stimulating hormone; Ne: negative; Po: positive; case: women or partner with TB; control: healthy women with healthy partner.

**Table 2:** The outcomes of pregnancy in cases and controls.

Variables		Control (31945)	Case (6389)	*P*
Gestational weeks		39.00 [38.00, 40.00]	39.00 [38.00, 40.00]	<0.001
Premature birth	No	31326 (98.1)	6233 (97.6)	0.01
	Yes	619 (1.9)	156 (2.4)	
LBW	No	31453 (98.5)	6261 (98.0)	0.009
	Yes	492 (1.5)	128 (2.0)	
Stillbirth	No	31898 (99.9)	6369 (99.7)	0.006
	Yes	47 (0.1)	20 (0.3)	
Birth gender	Man	16443 (51.5)	3317 (51.9)	0.525
	Women	15502 (48.5)	3072 (48.1)	
Birth weight (g)		3260.00 [3000.00, 3500.00]	3250.00 [3000.00, 3500.00]	<0.001

LBW: low birth weight; the distribution of continuous variables is expressed as the median [lower quartile, upper quartile]; case: women or partner with TB; control: healthy women with healthy partner.

**Table 3:** Adjusted odds ratio estimates and 95% confidence intervals of PTB, stillbirth, and LBW for each group.

	PTB (adjusted OR 95% CI)^a^	Stillbirth (adjusted OR 95% CI)^b^	LBW (adjusted OR 95% CI)^c^
Women or partner with TB	0.97 (0.80-1.16)	1.89 (1.09-3.16)	0.97 (0.79-1.19)
TB women with healthy partner	0.87 (0.66-1.12)	1.48 (0.61-3.09)	1.04 (0.79-1.35)
Healthy women with TB partner	1.03 (0.81-1.29)	2.13 (1.10-3.86)	0.89 (0.67-1.16)

PTB: preterm birth; LBW: low birth weight; OR: odds ratio; CI: confidence interval. ^a^Model adjusted for female's characteristics (age, ethnicity, education level, intensity of work, residence, has regular menstrual cycle, dysmenorrhea, gravidity, eat meat and eggs regularly, passive smoking, drinking, pressure of life and work, BMI, and creatinine), history of diseases in female (anemia, history of premature birth, history of natural abortion, and history of artificial abortion), and husband's characteristics (age, ethnicity, education level, intensity of work, eat raw meat, smoking, passive smoking, drinking, pressure of life and work, SBP (mmHg), blood group, and ALT). ^b^Model adjusted for female's characteristics (ethnicity and dysmenorrhea), history of stillbirth, and husband's characteristics (age, ethnicity, SBP, and DBP). ^c^Model adjusted for female's characteristics (age, ethnicity, education level, intensity of work, residence, has regular menstrual cycle, menstrual blood volume, dysmenorrhea, passive smoking, drinking, pressure of life and work, BMI, and creatinine), history of diseases in female (anemia, history of premature birth, and history of natural abortion), and husband's characteristics(age, education level, intensity of work, eat raw meat, smoking, passive smoking, drinking, pressure of life and work, SBP, blood group, and ALT).

## Data Availability

The datasets used and analyzed during the current study are available from the corresponding authors on reasonable request.

## Supplementary Materials

Supplementary 1 Table S1 comparison of characteristics and pregnancy outcomes among couples with different conditions of TB.

Supplementary 2 Table S2 adjusted odds ratio estimates and 95% confidence intervals of PTB, stillbirth, and LBW in pregnant women without TB treatment.
