# Supplementary material for: Increased Risk of Stillbirth among Women whose Partner Has Tuberculosis
Source: Biomed Res Int. 2021 Sep 14;2021:1837881. doi: 10.1155/2021/1837881 (PMC8459173; doi:10.1155/2021/1837881)
Supplement: Supplementary 1 — Table S1 comparison of characteristics and pregnancy outcomes among couples with different conditions of TB. [file 1837881.f1.pdf]

Table S1. Comparison of characteristics and pregnancy outcomes among couples with different condition of TB

Variables		Women with TB				Women without TB		
		Partner with TB (55)	P^{a}	Partner without TB (2859)	P^{b}	Partner with TB ((3475)	P^{c}	Partner without TB (31945)
**Female's characteristics**								
Age (year)		29.00 [27.00, 32.00]	0.068	28.00 [26.00, 31.00]	0.167	28.00 [25.00, 31.00]	0.177	28.00 [25.00, 31.00]
Ethnicity	Han	42 (76.4)	<0.001	2564 (89.7)	<0.001	3146 (90.5)	0.002	29398 (92.0)
	Others	13 (23.6)		295 (10.3)		329 (9.5)		2547 (8.0)
Education level	Junior high school and below	15 (27.3)	0.002	957 (33.5)	<0.001	1092 (31.4)	<0.001	15471 (48.4)
	High school and junior college	32 (58.2)		1620 (56.7)		2059 (59.3)		14926 (46.7)
	Bachelor and above	8 (14.5)		282 (9.9)		324 (9.3)		1548 (4.8)
Intensity of work	Light	18 (32.7)	0.001	983 (34.4)	<0.001	1179 (33.9)	<0.001	6515 (20.4)
	Moderate	16 (29.1)		623 (21.8)		762 (21.9)		4492 (14.1)
	Heavy	21 (38.2)		1253 (43.8)		1534 (44.1)		20938 (65.5)
Residence	Country	38 (69.1)	0.062	1947 (68.1)	<0.001	2354 (67.7)	<0.001	26348 (82.5)
	City	17 (30.9)		912 (31.9)		1121 (32.3)		5597 (17.5)
Menarche age		14.00 [12.50, 15.00]	0.887	13.00 [13.00, 14.00]	0.756	13.00 [13.00, 14.00]	0.547	14.00 [13.00, 14.00]
Is regular of menstruas cycle	No	7 (12.7)	0.085	281 (9.8)	<0.001	367 (10.6)	<0.001	1729 (5.4)
	Yes	48 (87.3)		2578 (90.2)		3108 (89.4)		30216 (94.6)
Menstrual blood volume	Much	3 (5.5)	<0.001	89 (3.1)	<0.001	117 (3.4)	<0.001	819 (2.6)
	Median	43 (78.2)		2459 (86.0)		3035 (87.3)		29815 (93.3)
	Few	9 (16.4)		311 (10.9)		323 (9.3)		1311 (4.1)
Dysmenorrhea	No	26 (47.3)	0.003	1538 (53.8)	<0.001	1964 (56.5)	<0.001	22068 (69.1)
	Yes	29 (52.7)		1321 (46.2)		1511 (43.5)		9877 (30.9)
Gravidity	0	18 (32.7)	0.067	1177 (41.2)	<0.001	1528 (44.0)	<0.001	14970 (46.9)
	1	22 (40.0)		900 (31.5)		1085 (31.2)		11946 (37.4)

	>= 2	15 (27.3)		782 (27.4)		862 (24.8)		5029 (15.7)
Parity	0	33 (60.0)	0.449	1703 (59.6)	<0.001	2051 (59.0)	<0.001	16916 (53.0)
	>= 1	22 (40.0)		1156 (40.4)		1424 (41.0)		15029 (47.0)
Eat meat and eggs regularly	NO	0 (0.0)	1	29 (1.0)	0.47	33 (0.9)	0.675	276 (0.9)
	Yes	55 (100.0)		2830 (99.0)		3442 (99.1)		31669 (99.1)
Anorexia vegetables	NO	55 (100.0)	1	2848 (99.6)	0.223	3446 (99.2)	0.089	31759 (99.4)
	Yes	0 (0.0)		11 (0.4)		29 (0.8)		186 (0.6)
Eat raw meat regularly	NO	55 (100.0)	1	2834 (99.1)	0.002	3441 (99.0)	<0.001	31806 (99.6)
	Yes	0 (0.0)		25 (0.9)		34 (1.0)		139 (0.4)
Smoking	NO	55 (100.0)	1	2835 (99.2)	<0.001	3445 (99.1)	<0.001	31854 (99.7)
	Yes	0 (0.0)		24 (0.8)		30 (0.9)		91 (0.3)
Passive smoking	NO	37 (67.3)	0.001	2028 (70.9)	<0.001	2531 (72.8)	<0.001	27747 (86.9)
	Yes	18 (32.7)		831 (29.1)		944 (27.2)		4198 (13.1)
Drinking	NO	50 (90.9)	0.253	2584 (90.4)	<0.001	3138 (90.3)	<0.001	30697 (96.1)
	Yes	5 (9.1)		275 (9.6)		337 (9.7)		1248 (3.9)
Pressure of life and work	Never	33 (60.0)	0.001	1726 (60.4)	<0.001	2068 (59.5)	<0.001	24511 (76.7)
	Mild	6 (10.9)		411 (14.4)		505 (14.5)		3994 (12.5)
	Moderate	13 (23.6)		629 (22.0)		788 (22.7)		3166 (9.9)
	Severe	3 (5.5)		93 (3.3)		114 (3.3)		274 (0.9)
BMI (Kg/m^2)		20.89 [19.29, 23.00]	0.6	20.32 [18.83, 22.26]	<0.001	20.81 [19.14, 22.95]	<0.001	20.57 [19.04, 22.67]
SBP (mmHg)		110.00 [101.50, 115.00]	0.684	109.00 [100.00, 115.00]	0.029	110.00 [101.00, 117.00]	0.005	110.00 [100.00, 115.00]
DBP (mmHg)		70.00 [66.50, 77.00]	0.435	70.00 [65.00, 76.00]	0.054	70.00 [65.00, 76.00]	0.581	70.00 [66.00, 75.00]
Blood group	O	22 (40.0)	0.723	1040 (36.4)	0.007	1308 (37.6)	<0.001	10736 (33.6)
	A	16 (29.1)		803 (28.1)		988 (28.4)		9279 (29.0)
	B	14 (25.5)		802 (28.1)		922 (26.5)		9122 (28.6)
	AB	3 (5.5)		214 (7.5)		257 (7.4)		2808 (8.8)
Rh blood group	Po	55 (100.0)	1	2852 (99.8)	0.611	3466 (99.7)	0.657	31843 (99.7)
	Ne	0 (0.0)		7 (0.2)		9 (0.3)		102 (0.3)
FBG (mmol/L)		4.70 [4.30, 5.03]	0.177	4.80 [4.40, 5.21]	0.037	4.82 [4.41, 5.21]	<0.001	4.78 [4.32, 5.20]
ALT (U/L)		14.00 [11.00, 23.85]	0.64	14.90 [11.00, 20.00]	<0.001	15.00 [11.00, 22.00]	<0.001	16.00 [11.70, 22.90]
Creatinine (μmol/L)		60.00 [51.00, 65.65]	0.054	59.00 [51.00, 70.00]	<0.001	59.00 [51.00, 69.30]	<0.001	63.00 [53.00, 74.00]
TSH (mIU/L)		1.90 [1.26, 2.78]	0.099	1.81 [1.17, 2.65]	<0.001	1.76 [1.14, 2.66]	<0.001	1.61 [1.07, 2.34]

**Hostory of diseases in female**								
Anemia	No	54 (98.2)	1	2787 (97.5)	0.014	3292 (94.7)	<0.001	31355 (98.2)
	Yes	1 (1.8)		72 (2.5)		183 (5.3)		590 (1.8)
Thyroid disease	No	54 (98.2)	0.943	2828 (98.9)	0.009	3398 (97.8)	<0.001	31739 (99.4)
	Yes	1 (1.8)		31 (1.1)		77 (2.2)		206 (0.6)
History of premature birth	No	55 (100.0)	1	2847 (99.6)	0.114	3462 (99.6)	0.207	31867 (99.8)
	Yes	0 (0.0)		12 (0.4)		13 (0.4)		78 (0.2)
History of stillbirth	No	55 (100.0)	1	2814 (98.4)	<0.001	3425 (98.6)	<0.001	31693 (99.2)
	Yes	0 (0.0)		45 (1.6)		50 (1.4)		252 (0.8)
History of natural abortion	No	52 (94.5)	0.766	2687 (94.0)	<0.001	3217 (92.6)	<0.001	30929 (96.8)
	Yes	3 (5.5)		172 (6.0)		258 (7.4)		1016 (3.2)
History of artificial abortion	1	29 (52.7)	<0.001	1859 (65.0)	<0.001	2466 (71.0)	<0.001	26664 (83.5)
	2	18 (32.7)		626 (21.9)		680 (19.6)		3605 (11.3)
	3	8 (14.5)		374 (13.1)		329 (9.5)		1676 (5.2)
**Partner's characteristics**								
Age (year)		31.00 [28.50, 35.00]	0.003	30.00 [27.00, 33.00]	<0.001	30.00 [27.00, 34.00]	<0.001	29.00 [26.00, 33.00]
Ethnicity	Han	48 (87.3)	0.264	2606 (91.2)	0.013	3158 (90.9)	0.001	29537 (92.5)
	Other	7 (12.7)		253 (8.8)		317 (9.1)		2408 (7.5)
Education level	Junior high school and below	16 (29.1)	0.013	940 (32.9)	<0.001	1036 (29.8)	<0.001	15187 (47.5)
	High school and junior college	32 (58.2)		1635 (57.2)		2110 (60.7)		15185 (47.5)
	Bachelor and above	7 (12.7)		284 (9.9)		329 (9.5)		1573 (4.9)
Intensity of work	Light	20 (36.4)	<0.001	771 (27.0)	<0.001	956 (27.5)	<0.001	4974 (15.6)
	Moderate	13 (23.6)		741 (25.9)		897 (25.8)		5454 (17.1)
	Heavy	22 (40.0)		1347 (47.1)		1622 (46.7)		21517 (67.4)
Eat meat and eggs regularly	NO	1 (1.8)	1	42 (1.5)	0.027	43 (1.2)	0.244	323 (1.0)
	Yes	54 (98.2)		2817 (98.5)		3432 (98.8)		31622 (99.0)
Anorexia vegetables	NO	55 (100.0)	1	2835 (99.2)	0.085	3414 (98.2)	<0.001	31765 (99.4)
	Yes	0 (0.0)		24 (0.8)		61 (1.8)		180 (0.6)
Eat raw meat	NO	55 (100.0)	1	2816 (98.5)	<0.001	3428 (98.6)	<0.001	31734 (99.3)

	Yes	0 (0.0)		43 (1.5)		47 (1.4)		211 (0.7)
Smoking	NO	39 (70.9)	1	1778 (62.2)	<0.001	2365 (68.1)	<0.001	22878 (71.6)
	Yes	16 (29.1)		1081 (37.8)		1110 (31.9)		9067 (28.4)
Passive smoking	No	33 (60.0)	0.182	1596 (55.8)	<0.001	1858 (53.5)	<0.001	23041 (72.1)
	Yes	22 (40.0)		1263 (44.2)		1617 (46.5)		8904 (27.9)
Drinking	No	28 (50.9)	0.027	1461 (51.1)	<0.001	1777 (51.1)	<0.001	22007 (68.9)
	Yes	27 (49.1)		1398 (48.9)		1698 (48.9)		9938 (31.1)
Pressure of life and work	Never	27 (49.1)	<0.001	1661 (58.1)	<0.001	1801 (51.8)	<0.001	23371 (73.2)
	Mild	7 (12.7)		426 (14.9)		527 (15.2)		4723 (14.8)
	Moderate	19 (34.5)		654 (22.9)		954 (27.5)		3370 (10.5)
	Severe	2 (3.6)		118 (4.1)		193 (5.6)		481 (1.5)
BMI (Kg/m^2)		22.12 [19.72, 24.22]	0.022	23.04 [20.90, 25.47]	0.084	22.04 [20.13, 24.22]	<0.001	22.86 [21.00, 25.05]
SBP (mmHg)		118.00 [110.00, 122.00]	0.871	118.00 [110.00, 125.00]	<0.001	117.00 [110.00, 124.00]	0.051	118.00 [110.00, 121.00]
DBP (mmHg)		74.00 [68.00, 80.00]	0.95	75.00 [70.00, 80.00]	0.006	75.00 [70.00, 80.00]	0.944	75.00 [70.00, 80.00]
Blood group	O	16 (29.1)	0.666	1020 (35.7)	0.039	1299 (37.4)	<0.001	10861 (34.0)
	A	20 (36.4)		859 (30.0)		991 (28.5)		9280 (29.0)
	B	15 (27.3)		754 (26.4)		936 (26.9)		9003 (28.2)
	AB	4 (7.3)		226 (7.9)		249 (7.2)		2801 (8.8)
Rh blood group	Po	55 (100.0)	1	2857 (99.9)	0.025	3463 (99.7)	1	31839 (99.7)
	Ne	0 (0.0)		2 (0.1)		12 (0.3)		106 (0.3)
ALT (U/L)		26.00 [18.70, 37.00]	0.761	27.00 [18.00, 39.95]	<0.001	24.00 [16.00, 36.95]	0.008	25.00 [17.60, 36.00]
Creatinine (μmol/L)		82.00 [71.00, 89.40]	0.489	81.50 [73.00, 91.50]	0.387	81.00 [72.00, 91.91]	0.771	81.00 [72.40, 91.00]
**Outcomes**								
Gestational weeks		39.00 [38.00, 40.00]	0.56	39.00 [38.00, 40.00]	<0.001	39.00 [38.00, 40.00]	<0.001	39.00 [38.00, 40.00]
Premature birth	No	55 (100.0)	0.557	2795 (97.8)	0.298	3383 (97.4)	0.006	31326 (98.1)
	Yes	0 (0.0)		64 (2.2)		92 (2.6)		619 (1.9)
LBW	No	55 (100.0)	0.677	2795 (97.8)	0.006	3411 (98.2)	0.198	31453 (98.5)
	Yes	0 (0.0)		64 (2.2)		64 (1.8)		492 (1.5)
Stillbirth	No	55 (100.0)	1	2852 (99.8)	0.306	3462 (99.6)	0.004	31898 (99.9)
	Yes	0 (0.0)		7 (0.2)		13 (0.4)		47 (0.1)
Birth gender	Man	24 (43.6)	0.298	1489 (52.1)	0.546	1804 (51.9)	0.634	16443 (51.5)
	Women	31 (56.4)		1370 (47.9)		1671 (48.1)		15502 (48.5)

Birth weight (g)	3250.00 [3000.00, 3500.00]	0.472	3250.00 [3000.00, 3500.00]	0.002	3250.00 [3000.00, 3500.00]	0.002	3260.00 [3000.00, 3500.00]

b: *P* values for comparison between healthy women with TB partner and group healthy women with healthy partner;
c: *P* values for comparison between TB women with healthy partner and group healthy women with healthy partner;
a: *P* values for comparison between TB women with TB partner and group healthy women with healthy partner;
The distribution of continuous variables is expressed as the median [lower quartile, upper quartile]; BMI: body mass index; SBP: systolic blood pressure; DBP: diastolic blood pressure; FBG: fasting blood glucose; ALT: alanine aminotransferase; TSH: thyroid stimulating hormone; Ne: negative; Po: positive; LBW: low birth weight; TB: Tuberculosis
