# Supplementary material for: Increased Risk of Stillbirth among Women whose Partner Has Tuberculosis
Source: Biomed Res Int. 2021 Sep 14;2021:1837881. doi: 10.1155/2021/1837881 (PMC8459173; doi:10.1155/2021/1837881)
Supplement: Supplementary 2 — Table S2 adjusted odds ratio estimates and 95% confidence intervals of PTB, stillbirth, and LBW in pregnant women without TB treatment. [file 1837881.f2.pdf]

Table S2 Adjusted odds ratio estimates and 95% confidence intervals of PTB, stillbirth and LBW in pregnant women without TB treatment

	PTB (Adjusted OR 95% CI) [a]	Stillbirth (Adjusted OR 95% CI) [b]	LBW (Adjusted OR 95% CI) [c]
Women or partner with TB	0.97 (0.80-1.17)	1.89 (1.09-3.17)	0.97 (0.78-1.18)
TB women with healthy partner	0.87 (0.66-1.13)	1.50 (0.61-3.12)	1.03 (0.78-1.35)
Healthy women with TB partner	1.03 (0.81-1.29)	2.13 (1.10-3.86)	0.89 (0.67-1.16)

PTB: preterm birth; LBW: low birth weight; OR: odds ratio; CI: confidence interval;

a: model adjusted for female's characteristics (age, ethnicity, education level, intensity of work, residence, is regular of menstruas cycle, dysmenorrhea, gravidity, eat meat and eggs regularly, passive smoking, drinking, pressure of life and work, BMI and creatinine), history of diseases in female (anemia, history of premature birth, history of natural abortion and history of artificial abortion) and husband's characteristics (age, ethnicity, education level, intensity of work, eat raw meat, smoking, passive smoking, drinking, pressure of life and work, SBP (mmHg), blood group and ALT)

b: model adjusted for female's characteristics (ethnicity and dysmenorrhea), history of stillbirth and husband's characteristics (age, ethnicity, SBP and DBP)

c: model adjusted for female's characteristics (age, ethnicity, education level, intensity of work, residence, is regular of menstruas cycle, menstrual blood volume, dysmenorrhea, passive smoking, drinking, pressure of life and work, BMI and creatinine), history of diseases in female (anemia, history of premature birth and history of natural abortion) and husband's characteristics(age, education level, intensity of work, eat raw meat, smoking, passive [illegible]
